# A thick placenta: a predictor of adverse pregnancy outcomes

**DOI:** 10.1186/2193-1801-3-353

**Published:** 2014-07-11

**Authors:** Ichiro Miwa, Masakatsu Sase, Mayumi Torii, Hiromi Sanai, Yasuhiko Nakamura, Kazuyuki Ueda

**Affiliations:** Department of Obstetrics and Gynecology, Yamaguchi Grand Medical Center, 77 Oosaki, Houhu, 747-8511 Japan

**Keywords:** Thick placenta, Measurement of placental thickness, Perinatal outcome, Ultrasonography

## Abstract

**Purpose:**

The aim of this study is to evaluate the efficacy of an ultrasonographic measurement of placental thickness and the correlation of a thick placenta with adverse perinatal outcome.

**Methods:**

Placental thickness was measured in single gravidas, 16 to 40 weeks of gestation, between 2005 and 2009. Placentas were considered to be thick if their measured thickness were above the 95th percentile for gestational age.

**Results:**

The incidence of thick placentas was 4.3% (138/3,183). Perinatal morbidity and neonatal conditions were worse in cases with thick placenta rather than without thick placenta.

**Conclusions:**

Ultrasonographic measurement of placental thickness is a simple method to estimate placental size. Thick placenta may be a useful predictor of adverse pregnancy outcomes.

## Introduction

The placenta is an important organ, maintaining fetal growth and well-being. In 1979, Grannum et al. (1979) first described a relation between ultrasonographic findings of the placenta and fetal maturity in high-risk pregnancies. Further researches revealed that morphological findings, such as echogenesity of the placenta (Quinlan et al. 1982), sonolucent placental lakes (Haney and Trought 1980; Jauniaux et al. 1994; Thompson et al. 2002), and thick placenta (Thompson et al. 2002), exhibited a poor correlation with perinatal outcome. However, in several reports (Jauniaux et al. 1994; Wolf et al. 1989; Elchalal et al. 2000; Dombrowski et al. 1992; Raio et al. 2004), placental size, as measured by various ultrasonographic methods, is a useful predictor of adverse pregnancy outcome.

Here we evaluate the efficacy of a simple ultrasonographic method of measuring placental thickness and the correlation of a thick placenta with adverse perinatal outcome.

## Materials and methods

This is a retrospective observational study. We reviewed the records of single gravidas who underwent at least one ultrasonographic examination between 16 and 40 weeks of gestation and delivered at Yamaguchi Grand Medical Center, Japan, from 2005 to 2009. Gestational age was determined by reliable recollection of the last menstrual period and confirmed by an ultrasonographic examination within 14 weeks of gestation in all cases. This study was approved by the institutional ethics committee of the hospital.

**Figure 1:**
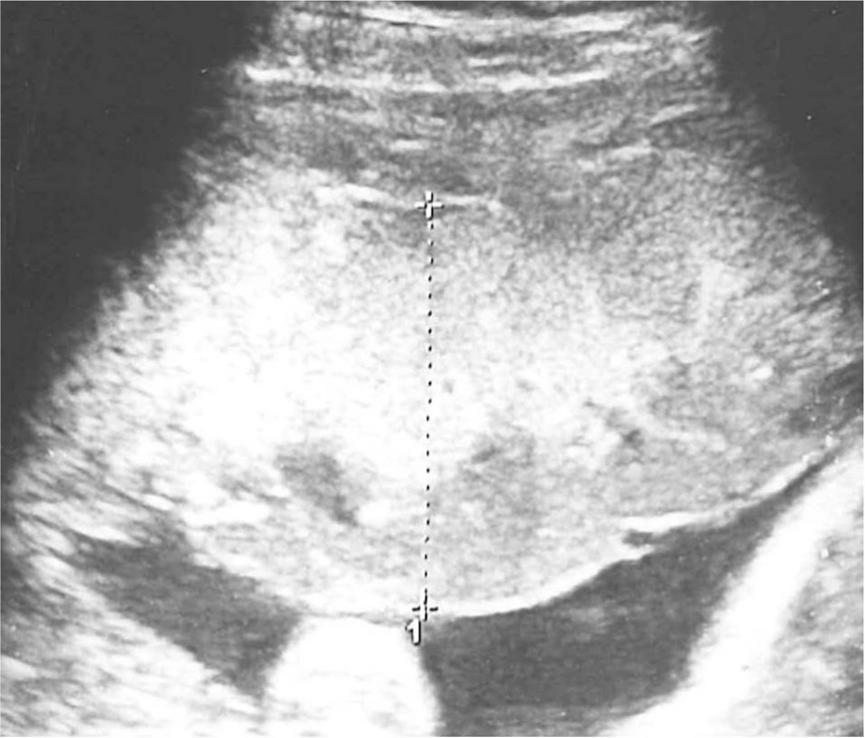
**Placental thickness was measured at the thickest portion of the placenta or beneath the cord insertion, and in a representative portion perpendicular to the chorionic plate.**

Ultrasonographic examinations were performed with a ProSound a10 fitted with a 3.5 MHz transducer (Aloka Medical). If patients underwent multiple examinations during their pregnancies, the first sets of measurements were used in this study. Fetal biparietal diameter, abdominal circumference, and femur length were measured. Pulsed Doppler evaluation of the umbilical artery, middle cerebral artery, ductus venosus, and uterine arteries was conducted. Placental thickness was measured at the thickest portion of the placenta or beneath the cord insertion, and in a representative portion perpendicular to the chorionic plate (Dombrowski et al. 1992) (Figure 1). Placentas were determined to be thick if the thickness value was above the 95th percentile for gestational age (Jauniaux et al. 1994).

Perinatal outcome was assessed based upon gestational age at delivery, birth weight, Apgar scores, pH of the umbilical artery, number of emergency cesarean section deliveries, non-reassuring fetal status (NRFS), fetal growth restriction (FGR), intrauterine fetal demise (IUFD), abruptio placentae, pregnancy-induced hypertension (PIH), gestational diabetes mellitus (GDM), and congenital anomalies.

Statistical analyses were performed using the Statistical Package for the Social Sciences (SPSS) Statistics 13.0 software (IBM, Armonk, NY). The unpaired *t*-test and *X*^2^ test were used, as appropriate. A value of P < 0.05 was considered significant.

## Results

**Figure 2:**
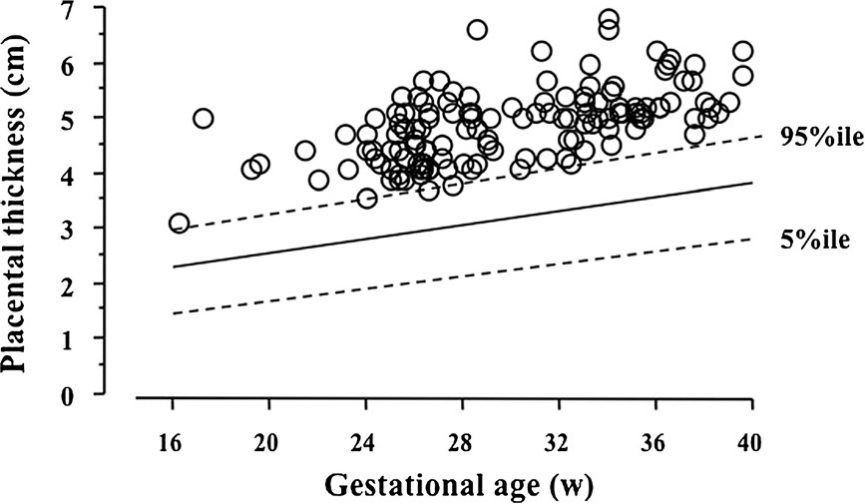
**Placentas were diagnosed as thick if they were above the 95th percentile for gestational age.** Between 16 and 40 weeks of gestation, 4.3% (n = 138) of patients were diagnosed with thick placentas.

A total of 3,183 subjects met the study criteria. Of these, 4.3% (n = 138) had thick placentas, as determined between 16 and 40 weeks of gestation (Figure 2).

Gestational age at delivery was earlier and birth weight was smaller in the cases with thick placenta than in those without thick placenta (Table 1). The values of Apgar score at 1 minute and pH of umbilical artery in the cases with thick placenta were also significantly lower than in those without thick placenta (Table 1). There is no difference in the Pulsed Doppler evaluation of the fetal and maternal blood flow waveforms in two groups.

**Table 1:** **Clinical characteristics of the two groups**

	Thick placentas (n = 138)	Controls (n = 3045)	p value
Maternal age	30 (16–43)	30 (15–45)	N.S.
Gestational age at delivery	38 (27–41)	39 (23–42)	< 0.05
Birth weight	2882 (710–3884)	2956 (312–4410)	< 0.05
1-min Apgar score	8 (0–9)	8 (0–10)	< 0.05
5-min Apgar score	9 (0–10)	9 (0–10)	N.S.
pH of umbilical artery	7.312 (6.989–7.405)	7.314 (6.547–7.544)	< 0.05

Median (range). N.S., not significant.

Perinatal morbidity and neonatal conditions were worse in cases associated with thick placentas (Table 2). The rates of emergency cesarean section deliveries, NRFS, FGR, abruptio placentae, PIH, and congenital anomalies were significantly higher in cases with thick placenta than without thick placenta (Table 2). No significant difference was observed in IUFD and GDM rates.

**Table 2:** **The incidence of complications and perinatal morbidity**

	Thick placentas (n = 138)	Controls (n = 3045)	p value
eC/S	31 (22.5%)	410 (13.5%)	< 0.05
NRFS	42 (30.4%)	458 (15.0%)	< 0.05
SGA	28 (20.3%)	371 (12.2%)	< 0.05
IUFD	1 (0.7%)	12 (0.4%)	N.S.
Abruptio placentae	4 (2.9%)	29 (1.0%)	< 0.05
PIH	14 (10.1%)	86 (2.8%)	< 0.05
GDM	3 (2.2%)	23 (0.8%)	N.S.
Congenital anomalies	13 (9.4%)	96 (3.2%)	< 0.05

Number (%). N.S., not significant. eCS, emergency cesarean section.

NRFS, non-reassuring fetal status. SGA, small for gestational.

IUFD, intrauterine fetal death. PIH, pregnancy induced hypertension.

GDM, gestational diabetes mellitus.

## Discussion

Previous reports described an association of thick placentas with elevated risk of adverse perinatal outcome, e.g. abruptio placentae, admission in neonatal intensive care unit, congenital anomalies, perinatal death, fetal growth restriction (FGR), heavy-for-gestational-dates infant (HFD), pregnancy-induced hypertension (PIH), preterm birth gestational age, birth weight, Apgar scores, pH of the umbilical artery, number of emergency cesarean section deliveries, non-reassuring fetal status (NRFS), intrauterine fetal demise (IUFD), pregnancy-induced hypertension (PIH), and gestational diabetes mellitus (GDM) (Jauniaux et al. 1994; Elchalal et al. 2000; Dombrowski et al. 1992; Raio et al. 2004). Conversely, Thompson et al. (2002) found no correlation between a thick placenta and poor obstetrical outcome, apart from a mild association with severe preeclampsia. The present study shows that thick placenta, as determined by ultrasonographic measurement, is associated with abruptio placentae, pregnancy-induced hypertension (PIH), non-reassuring fetal status (NRFS), fetal growth restriction (FGR), low Apgar scores, low pH of the umbilical artery, and number of emergency cesarean section deliveries. These abnormalities are closely related with placental dysfunction. Indeed, placental infarction, intervillous thrombosis, and inflammation were often detected in thick placenta by pathological examination (Jauniaux et al. 1994; Elchalal et al. 2000; Raio et al. 2004). Placental dysfunction may also result in thick placenta by the compensatory proliferation and edema of placental villi (Raio et al. 2004; Fox 1978).

An association between placental volume measured with ultrasound and perinatal outcome has already been reported (Jauniaux et al. 1994; de Paula et al. 2008). However, they required accurate measurement with computerized equipment, which is not yet widely available. In addition, these ultrasonographic methods are too complex. It is difficult to perform, as part of the current routine, antenatal ultrasonographic examination because they are very time consuming. Longitudinal ultrasonographic studies of placental volume have demonstrated a wide variation at each stage of gestation, from approximately 110–425 mL at 23 weeks to 340–1000 mL at term (Hellman et al. 1970; Bleker et al. 1977; Geirsson et al. 1985). The measurement of placental thickness is a simple method to estimate placental size *in utero*, and the distribution of values is narrow (Jauniaux et al. 1994). The prevalence of ultrasonographically thick placentas reported in the literature varies from 0.6% to 7.8% (Jauniaux et al. 1994; Thompson et al. 2002; Elchalal et al. 2000; Dombrowski et al. 1992). The present study found the prevalence of an ultrasonographically thick placenta to be 4.3%, within the standard reported range (Jauniaux et al. 1994). Thick placentas were found to be related to adverse prenatal outcomes. The neonatal conditions associated with thick placentas were also worse than those that in cases without thick placentas. However, the difference between the two groups was small and the median values were within the normal range. Fetal and maternal blood flow waveforms, representing a change in early stages of abnormal conditions (Arabin et al. 1992), were not different between the two groups at the time when placental thickness was detected. These facts indicate that the ultrasonographically thick placenta may represent a latent phase in placental dysfunction. Measurement of placental thickness is quite easy by conventional ultrasonographic equipment and it is possible to include the measurement into the routine examination. It may be a useful method to detect high risk pregnancies.

## Conclusion

The ultrasonographic measurement of placental thickness is a simple method to estimate placental size, and a thick placenta may be a useful predictor of adverse pregnancy outcomes.
